# Patient, carer and public involvement in major system change in acute stroke services: The construction of value

**DOI:** 10.1111/hex.12668

**Published:** 2018-01-18

**Authors:** Christopher McKevitt, Angus I.G. Ramsay, Catherine Perry, Simon J. Turner, Ruth Boaden, Charles D.A. Wolfe, Naomi J. Fulop

**Affiliations:** ^1^ School of Population Health & Environmental Sciences Faculty of Life Sciences and Medicine King's College London London UK; ^2^ Department of Applied Health Research University College London London UK; ^3^ Alliance Manchester Business School University of Manchester Manchester UK; ^4^ Centre for Primary Care Division of Population Health, Health Services Research and Primary Care School of Health Sciences Faculty of Biology, Medicine and Health University of Manchester Manchester UK

**Keywords:** impact, involvement, major system change, participation, stroke, value

## Abstract

**Background:**

Patient and public involvement is required where changes to care provided by the UK National Health Service are proposed. Yet involvement is characterized by ambiguity about its rationales, methods and impact.

**Aims:**

To understand how patients and carers were involved in major system changes (MSCs) to the delivery of acute stroke care in 2 English cities, and what kinds of effects involvement was thought to produce.

**Methods:**

Analysis of documents from both MSC projects, and retrospective in‐depth interviews with 45 purposively selected individuals (providers, commissioners, third‐sector employees) involved in the MSC.

**Results:**

Involvement was enacted through consultation exercises; lay membership of governance structures; and elicitation of patient perspectives. Interviewees’ views of involvement in these MSCs varied, reflecting different views of involvement per se, and of implicit quality criteria. The value of involvement lay not in its contribution to acute service redesign but in its facilitation of the changes developed by professionals. We propose 3 conceptual categories—agitation management, verification and substantiation—to identify types of process through which involvement was seen to facilitate system change.

**Discussion:**

Involvement was seen to have strategic and intrinsic value. Its strategic value lay in facilitating the implementation of a model of care that aimed to deliver evidence‐based care to all; its intrinsic value was in the idea of citizen participation in change processes as an end in its own right. The concept of value, rather than impact, may provide greater traction in analyses of contemporary involvement practices.

## 1 INTRODUCTION

Internationally, citizens, patients and family carer‐givers are increasingly being positioned as collaborators in the labour required to produce and maintain health.^1^, ^2^, ^3^, ^4^, ^5^ This includes involvement in individual patient care, research, and planning, development and improvement of health services.^6^, ^7^, ^8^ Carman et al^9^ argue that such involvement challenges a dominant paternalistic approach to health care and systems and has the potential to transform patients, improve outcomes through better systems of care and reduce health‐care costs. Yet involvement concepts and practices are characterized by ambiguity in terms of terminology,^10^, ^11^ rationales,^12^, ^13^ values^14^ and the attributes of those who are invited or choose to be “involved”.^13^, ^15^ The literature also suggests an anxiety that involvement aspirations are not being met as involvement practices are tokenistic rather than meaningful.^16^, ^17^, ^18^, ^19^ What makes involvement meaningful or tokenistic has hardly been explained, but failure to involve in a “meaningful” way has been attributed to the different types of knowledge associated with experts and lay people,^20^ and the privileging of expert over experiential knowledge.^21^ Involvement methods used have been criticized for their failure to adopt democratic models,^16^ ensuring that traditional models of decision making are maintained.^17^ Evidence that the promissory benefits of involvement in research and service development have been realized is limited, raising questions about not just how to evaluate impact but what impacts should be considered.^22^, ^23^, ^24^

In this paper, we consider the practices and achievements of patient and public involvement in major system change (MSC) that aimed to improve the delivery of acute stroke services in 2 metropolitan cities, Greater Manchester (GM) and London in England. We draw on data from an evaluation of these MSCs that included investigation of how the requirement to involve patients, carers and the public^25^ was put into practice; and how involvement and its value were represented in interviews with those involved in service redesign. The meanings ascribed to involvement, we will argue, lay not in the diverse values thought to underpin involvement^14^ but in the production of value arising from performances of involvement.

## 2 MAJOR SYSTEM CHANGE

Major system change refers to large‐scale reorganization of health services to create new care pathways and sometimes involving centralization of services with a reduction in the number of provider units.^26^ The aim is to improve care quality by making the most efficient use of resources. Inevitably, MSC involves multiple stakeholders across different constituencies, with different interests.

A realist review of MSC initiatives identified 5 “simple rules” underpinning successful initiatives.^27^ These were related to style of leadership, development of feedback loops, learning from past experience and engagement of physicians. Rule 5 recommends the inclusion of patients and families, arguing “It is perhaps self‐evident that the more service users that are involved in the change process, the more “patient centered” the services will become” (Best et al 2012, 441). We have previously proposed a more nuanced version of this “rule” that calls attention to how the various drivers of MSC can influence the weight of different stakeholders’ perspectives that may give rise to tension between patients’ and others’ perspectives.^28^

A rapid systematic review of studies investigating public engagement (described as synonymous with involvement) in planning MSC interventions^3^ reported that there is no agreed definition of involvement/engagement; a wide range of activities are used; studies have reported impact in terms of process but not outcome; and that it is not possible to isolate how engagement affects decision making or the kinds of MSCs that are implemented. The review called for further research to identify the impact of involvement in MSC and to isolate the most impactful methods of involving publics.

### 2.1 MSC in stroke care

**Figure 1:**
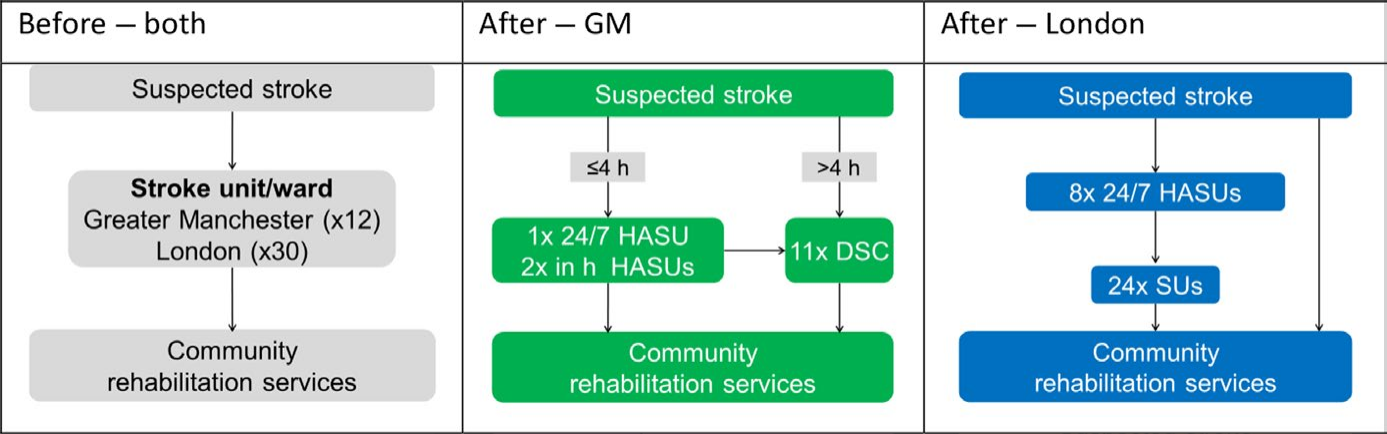
Care pathways before and after major system change

There have been significant advances in the evidence base of effective stroke care,^29^, ^30^ and concerted policy drives to improve the quality of care. Yet national audit data suggested ongoing shortfalls in the delivery of best‐quality acute care, with concerns about the proportions of patients being treated in a stroke unit or receiving clot‐busting treatment (thrombolysis) where indicated. In London and GM, networks of clinicians and senior health service managers independently undertook to re‐organize stroke services to improve access to acute stroke care. They devised and in 2010 implemented 2 different models of the acute care pathway, both of which established hyperacute stroke units (HASUs) providing care over the first 72 hours after stroke, including rapid assessment by specialized stroke medical teams, brain imaging and, if appropriate, thrombolysis (clot‐busting therapy). Patients were thereafter to be discharged or transferred to their local stroke unit for further treatment. In GM, only patients presenting within 4 hours of stroke would be admitted to a HASU; in London, all cases of suspected stroke were to be taken to HASU. Figure 1 presents the standard acute care pathway before MSC and the different pathways implemented in London and GM.

These were significant changes involving multiple organizations and large numbers of patients. In London, the previous acute stroke care system involved 31 local commissioning organizations, 32 providers and the local ambulance service. In GM, the system involved 10 commissioning organizations, 9 providers and the ambulance service. In both areas, multiple agencies oversaw the development of and negotiations for the new centralized services over at least 2 years, with the work overseen by a specially instituted project boards, whose membership included providers, commissioners and patient representatives. Within 2 years of implementing the new centralized models of care, ˃8000 stroke patients were admitted to acute services in GM and 15 000 in London. There were concerns about the service change from the outset. Hospitals and professionals were concerned about implications of the new models of care in terms of loss of resources and in London the closure of 5 acute stroke wards. There were concerns about patient safety and acceptability since the new models meant journeys for some patients, beyond their locality, and transfers from one site to another. The need to engage all types of stakeholder was recognized if the proposed changes were to be implemented.

## 3 METHODS

Data for this study were collected during a retrospective evaluation that investigated how changes in the delivery and organization of acute stroke care were implemented and to what effects. We draw on project documents and data from in‐depth interviews with people involved in the implementation of the MSC. Interviews, which were conducted by (Author) and (Author) in 2012‐13, were designed to collect data on the entire process of change; in this paper, we focus specifically on participants’ accounts of how patient and public involvement was enacted during the process of preparing for and implementing the service changes.

Interviewees were purposively sampled to include a range of people involved in the MSC at national and local levels (Table 1); they provided informed written consent. Interviews used a topic guide designed for the study; they were audio recorded and professionally transcribed in full. A separate analysis was conducted to investigate involvement in MSC. All authors contributed to the data analysis, which was both inductive and deductive, as themes drew on both the a priori questions from the topic guide and those emerging from the data. Following initial coding of data, analysis sought to develop descriptive categories (for example of what kinds of involvement strategies were implemented) and then conceptual categories to propose a new interpretation of data. The findings, analysis and interpretation were refined in group discussions among AR, CP, SJT, NJF, RB and CM. Initial findings were also shared with the study advisory group to check for accuracy and the face validity of the interpretation being proposed.

**Table 1:** Profile of interviewees

Interviewees	London	GM	National	Total
Stroke network board	5	6	‐	11
Pan‐regional health authority	7	‐	‐	7
Service commissioners	3	1	‐	4
Service users or representatives	3	3	‐	6
Programme facilitation	2	1	‐	3
Clinical leads	2	1	‐	3
Provider organizations	2	1	‐	3
Stroke service staff	‐	3	‐	3
Ambulance service	1	1	‐	2
Local/national politicians	1	‐	2	3
Total	26	17	2	45

## 4 RESULTS

### 4.1 Participants

In‐depth interviews were conducted with 17 people in GM and 26 in London leading and involved at board level in the system change process, including, NHS commissioners and managers, clinicians delivering acute care in the 2 settings and employees of stroke voluntary sector organizations.

### 4.2 Involvement practices

Documentary analysis and interview data suggested that in both sites, multiple strategies were used to involve patients at a range of levels. Existing resources were accessed (such as NHS involvement managers and individual voluntary sector organizations); social and other media were used to invite patients to participate. In London, an involvement strategy was developed that outlined plans to access a range of public and patient groups and highlighted the need to access “hard‐to‐reach” groups. Interviewees in both areas identified different types of activity through which patients and public were involved as follows: stakeholder information and consultations events; lay membership of governance structures; and formal elicitation of patient perspectives post‐implementation. No similar single strategy document was developed in GM, but project documents, such as that detailing the governance framework, identified the organizational structures through which patient and public involvement, and public awareness and education would be developed and implemented.^31^

#### 4.2.1 Information and consultation events

In GM, 3 “stakeholder engagement events” were organized over 8 months between 2007 and 08, as service development plans were initiated and took shape. The aims of these events reflected the development of plans for service change. Initially, they sought to get agreement about the need for change, then to inform stakeholders about the planned changes and then to inform them about progress. Involvement was also described as making use of existing PPI structures—such as Stroke and Cardiac Networks and NHS Patient Advisory Liaison Services, and public health campaigning work into which information about the stroke service changes could be incorporated.

Consultations in London were much more extensive than in GM as these took place under the aegis of the larger regionwide review of health needs across a range of conditions and of current provisions of health care. The well‐funded consultation programme, Healthcare for London—Consulting the Capital,^32^ sought views of members of the public on the range of proposals put forward in the resulting document, Healthcare for London: A Framework for Action.^33^ The consultation process was developed, overseen and analysed by Ipsos MORI, and used a range of marketing and opinion‐seeking activities, including online media, presentations to lay and professional organizations, and consultation road shows at health authority level.^34^ The formal consultation received 4734 responses (in online, written, email and other formats) from a wide range of stakeholders that included not only individual members of the public but also local voluntary sector organizations, local council members, health and other professional bodies, and local politicians. Comparison between individuals who took part and the London population found some differences with over‐representation of women and older people; however, “the ethnic profile of respondents was broadly representative of Londoners” (Ipsos MORI 2009, 17).

The consultation had a clearly defined scope, concerning “Adult services for acute stroke care—explicitly the location and coverage of hyperacute services and acute services in London.” In response to the question about the proposal “to create more specialized centres for the treatment of severe injury, stroke and complex emergency surgery needs” 42% “strongly” agreed with the proposal to create more stroke specialist centres, and 25% tended to agree.^33^

A separate consultation process was subsequently held focusing on proposed changes for acute stroke and trauma service, at a cost of £1.2 million. Again, this entailed preparation of consultation materials, numerous public events and formal elicitation of views. It was estimated that participation in the consultation involved 14 000 individual visitors to the website, 13 000 visitors to health fairs and around 14 000 people attending meetings. Responses to the consultation document were received from 8100 individuals and 200 organizations. It was reported that 67% of respondents strongly agreed/tended to agree with the proposed model of acute stroke care that had been developed, that is that “about seven hospitals” across London should provide HASU care.^34^

#### 4.2.2 Membership of governance structures

Interviewees pointed to lay membership of governance bodies as a way in which involvement policy was implemented. In GM, a local activist (spouse‐carer of someone who had had a stroke in the past) and well known to professionals leading the service change was invited to join a working group and the Stroke Network Board, to which it reported. An employee of the Stroke Association and an NHS network PPI manager were also members of this Board. Similarly, in London, Stroke Association (a charity sector organization and service provider) employees sat on the project board and Clinical Expert panel which designed the service specifications to provide patient/carer representation. Additionally, a Stroke Patient and Carer subcommittee was established to discuss topics including approaches to PPI, the running of consultation events and the development of the new model of care, and to feedback to the user groups they represented. This subcommittee met 4 times during the life of the development process.

#### 4.2.3 Eliciting patient perspectives post‐implementation

Interviewees also identified collecting patient and carer views of the new service as a form of involvement. For example, 12 months into the delivery of the newly reconfigured acute stroke pathway in GM, a review was conducted that included a separate study using survey and a series of qualitative interviews to elicit patient and carer views of the new pathway. Stroke survivors were invited to take part via online and social media, and through contacts with and visits to support groups across the region. In total, 84 people (10% response rate) returned the questionnaire, largely expressing the view that being admitted to a specialist acute centre, rather than the local hospital was not a concern.

### 4.3 The quality of involvement

Our interviews did not ask participants to comment on whether involvement was meaningful or tokenistic, but many offered their own views of the quality of what was done, with wide variations in their appraisals.

Interviewees suggested that in GM there was little formal consultation of patients and the public. Public events were described as designed to provide information about the planned changes rather than to elicit views. For example, a commissioner said,

I don't think at any stage we said to the public of Greater Manchester, if there's such a thing – and there isn't – we didn't say, “Do you want three of these of five of these?” We never said that. (GM01)

In London, where there were sustained efforts to hold formal consultations 2 of 3 NHS managers interviewed were positive about the consultations because of the efforts made to encourage high levels of participation, and to be inclusive. An interviewee from the voluntary sector challenged this view, arguing consultation was not sufficiently accessible to people with stroke‐related aphasia, for example. Others suggested the consultation was useful because it secured buy‐in from the public or “political legitimacy.” However, one commissioner, who conceded that consultation was necessary because of societal expectations of transparency in public services, also expressed concerns about the limits of the public's knowledge to comment on proposed new plans. Others, including a commissioner and 2 doctors were either dubious, expressing doubt about the usefulness or validity of consultation, or considered it “a complete waste of time” (L02) because consulting the public had eclipsed the need to consult more widely with professionals providing long‐term services to stroke survivors.

There were also different evaluations of individuals’ contributions to governance structures. Several interviewees from GM spoke about the lone activist who was appointed to the project board as effective because of previous professional political experience, his ability in committee work, history as a campaigner for stroke service quality and even his challenging approach. Others rehearsed well‐known arguments about the limits of individual contributions because they were “self‐selected,” or the “usual suspects” and therefore unrepresentative or because they lacked experience in formal committee work.

The limits of how actively involved lay people could be in this involvement process were also recognized. For some, this was related to the nature of MSC itself. It required knowledge of a range of complex problems such as population needs, and resource implications and political implications. As the designs had been worked out by a core group of professionals, there was limited opportunity for lay people to influence the service design. For some interviewees, this meant that there was “no real involvement” while others conceded that while this might not be patient‐led involvement, consultation processes allowed patients to become “advocates for the model.”

The trope of the patient voice also figured in interviewees’ accounts. Involvement was seen as an opportunity for the patient voice to be articulated, represented by interviewees as an important corrective to the dominant perspectives of professionals and organizations. For example, a physician said:

People (professionals) have to, really have to be brought back to what's best for the patients and an awful lot of what gets discussed in the NHS is not about that, it's about what's best for my organisation. (GM05)

On the other hand, a minority of interviewees argued while patient voices may have been heard, they could not be acted on within the scope of the consultation activities which were specifically limited to the MSC projects’ redesign of acute stroke services. Nevertheless, these interviewees noted that patients took the opportunity to articulate another concern: the quality of rehabilitation services. A manager clearly made this point:

… every single question which was asked in the half hour or so that the meeting was thrown open for questions from the floor was about rehab […] Nobody asked a question or protested about the decisions we were making about, er, the hospital service but all the members of the public and their representative questions were about rehab. (L03)

Patients may have been content to leave acute service design to professionals who carefully made a convincing case for centralization as able to deliver better quality acute care for all. Yet the question of rehabilitation services was outside the brief of change leads, and even where involvement permitted dialogue between stakeholders, the question of rehabilitation and longer‐term care was inadmissible.

### 4.4 Constructing value

It could not therefore be argued that involvement in these examples of MSC influenced or improved the design of the acute stroke services. As we have previously argued, involvement was used instrumentally by programme leaders to gain support for change the case for which had already been made, and for service models already developed. For a minority of interviewees, this indicated a failure to achieve an ideal of patient‐led involvement, but for most, even if flawed, the practices of involvement had intrinsic value for the implementation of MSC. We identify 3 types of value the interview data suggest.

#### 4.4.1 Managing agitation

First, involvement was represented as a way of managing actual or potential resistance or agitation.^35^ Above, we report the significance attributed to a lone activist in GM who had a track record in agitating for improved stroke services, after his wife's own stroke. While interviewees recognized his expertise, he was also described as “grit in the system,” an irritant that produces change. In this case, it could be argued that there was an effort to manage an activist's agitation by incorporating him into official PPI structures.

At a broader level, we have already seen that MSC leads were concerned that patients and families might object to an acute care model that saw the patient being admitted to a non‐local hospital and care involving ambulance transfers from one hospital. Thus, involvement sought to anticipate and manage any dissent that might arise.

As Martin^36^ has argued, PPI could be a way of containing and managing citizen desires and action yet PPI itself might give rise to unanticipated forms of agitation, as happened in 2 localities in London, where local people and politicians objected to loss of local acute stroke services. Here, the MSC leads could point to the consultation work conducted across London as gathering “overwhelming support from everywhere else” (L03), thus trumping what could be portrayed as local interests. Indeed, the London model was consistently portrayed as pan‐London, rather than locality based.

#### 4.4.2 Verification

Involvement was also described as permitting what could be termed processes of verification that took place at different levels. Interviewees suggested that involvement permitted the pre‐empting of potential concerns patients and their family members might have about the fact that the new service might see them admitted to a specialist centre that was not their local hospital. This had been perceived as a potential source of disagreement or dissatisfaction among patients. As such, opposition remained limited, and MSC leads were able to verify that their proposed design was acceptable to patients. Secondly, interviewees cited examples of how lay people had been involved in the development of information materials. For example, the work in GM entailed development of information for ambulance crews instructing them on the new procedures for suspected stroke admissions. Patient input was sought here on the development of scripts to be used by ambulance crew taking patients to a specialist centre, rather than the local hospital. Similarly, interviewees reported that involvement processes enabled them to be reassured that MSC was the right way to proceed. For example, one interviewee reflected:

… it was really important to be able to have their voice, saying ‘This is a good thing. It should be done’. L03

#### 4.4.3 Substantiation

Finally, interviewees evoked an effect that we refer to as substantiation, making an idea physically present. Involvement processes enabled the service user simply to be present in the room (as Donaldson^37^ puts it) or at a public event. By being present, the service user embodied the “stroke patient,” as a representative in a symbolic sense rather than representative in any population/demographic sense in a way that was useful for several reasons. First, the physical presence of the patient relates to the normative status of involvement noted by previous authors: by being present in the room, patients provided physical evidence that involvement policy was being enacted. Thus, presence enabled demonstration of adherence to the NHS vision that health‐care development depends not just on “technocratic intervention and political whim but also upon social values pertaining to equality, inclusion and social justice” (Milewa^38^, 250). A stroke physician interviewed described the development of a mission statement at the outset of the work which asserted that the purpose of the MSC was to ensure equality of access to the best possible acute care for every stroke patient:

I thought we should be really clear what it is we were here for and we wrote it on a flip chart and it came out at every meeting and it got stuck up on the wall. Every citizen of Greater Manchester has equal access to high quality acute stroke care. And actually, that was really useful because when the arguments started you could then say, but how does that relate to our vision? (GM05)

Second, the patient's presence in the room was used to manage conflict between professionals faced with decisions that might have consequences for individuals, services or localities. The patient in the room was used to remind stakeholders that the ultimate goal was to improve the quality of patient care.

So… we're bringing the focus back to the patient… and what we're here for. We're not here to be arguing about politics and you know, who's the best stroke physician and, you know, peoples’ ego; it's about you know what would you want for your grandma or your mum if she had a stroke tomorrow. (GM13)

Whenever we had stakeholder events, you know, we would always have a speaker who was either a carer or someone from the stroke association or occasionally a patient… and again it meant right down at a kind of micro level, people remembered we were doing this to improve patient care, not to protect their institution or their profession. (L16)

Though seen as effective, it could also be argued that these instances of substantiation had the effect of reasserting the status of both patient and professional. The patient is recast as beneficiary of the work to improve quality of care, and the professional as expert provider of benefit. The traditional roles of patient and expert are maintained. In this way, the emancipatory vision of involvement as transforming roles through empowerment does not appear to have been realized.

## 5 DISCUSSION

This study drew on project documents and qualitative interviews with a wide range of professionals engaged in complex and protracted processes to redesign acute stroke care in 2 English cities. The case for MSC was constructed by professionals drawing on clinical and managerial experience, and examination of population‐level patient data that demonstrated the need to improve access to best evidence care. This was consistent with NHS strategic guidance that requires service‐level changes to be clinically led and underpinned by clinical evidence.^39^ This set the parameters of involvement from the outset: the case for change was professionally led, but the co‐operation and approval of a wide range of stakeholders including clinical staff, NHS managers and local politicians was required. Patient and public involvement was a tool to facilitate implementation of the changes. In this sense, involvement could be seen as instrumental, achieving the outcomes desired by professionals. However, we would further argue that rather than either “tokenistic”—suggesting a cynical position, or “meaningful”—implying conformity with some a priori agreed definition of what involvement means—involvement here was enacted in strategic ways.

Interviewees’ accounts varied widely in how they evaluated involvement. Consultations were seen as at worst a waste of time, to at best wide‐reaching, inclusive events in which the patient voice could be heard and professional transparency demonstrated. The contribution of individuals taking part in governance structures was also differently viewed. They were variously portrayed as powerful voices reminding professionals—at risk of promoting their own interests—of their true purpose; as making a limited contribution because of their limited competence in meeting behaviour; and as self‐selected and unrepresentative.

This implies that professionals controlled not only the agenda but also the manner in which involvement was enacted.^16^, ^40^ This may be inevitable. The model of involvement that dominates the NHS requires professionals to invite lay people to participate in activities which professionals design, focused on questions they identify. This differs from the involvement's political antecedents: self‐organizing patient movements, which drew on principles of social justice and emancipatory practice to challenge biomedical definitions of illness and solutions, counter‐discrimination and stigma, and call for action into emergent health problems.^41^ While involvement may promise a transformation in relations between patients and professionals, as Komporozos et al^42^ have argued, the ritual nature of PPI activities constitutes “a conservative form of engagement in health” (2016, 15) that serves to reinforce existing statuses, neutralizing the transformational potential of involvement.

Our data are limited in that they are retrospective, rather than contemporaneous accounts. The diversity of interviewees and their role in MSC means that each provides something of a partial view of which activities were undertaken and by whom. What emerges is a rather complex picture of diverse activities from information‐giving events through to research to collect accounts of patient experience—framed by participants as involvement. The data do not necessarily provide an accurate historical record of PPI in the 2 MSC projects, but they offer a moral account of implementing PPI in the projects.

Our study offers lessons for thinking about involvement in general and in relation to MSC. In particular, the findings represent a challenge to contemporary concerns that the literature reports processes of involvement but fails to report on impact and that improved methods to demonstrate impact are required.^3^, ^43^ Our interviewees’ accounts did not suggest that it was possible to demonstrate impact of involvement in a linear way, because involvement was not designed to effect but to support change. Involvement here was a strategically symbolic process that served to use the moral authority of the imagined but substantiated patient to support change implementation. Thus, involvement as enacted also reiterated the significance of involvement itself. Conklin et al^23^ and Li et al^24^ have suggested that rather than focus on impact, we should consider the quality of involvement in processual terms, either as democratic acts in their own right or strategic acts of informing and legitimizing.

Our study also finds limits to involvement as democratic process. The MSC sought to effect change in acute stroke care, and this set the parameters of what was admissible; consequently, patients’ concerns about the quality of care needed after discharge from hospital were rendered irrelevant. In London, the need to consider the whole stroke pathway was acknowledged by Health Care for London,^34^ but this acknowledgement was made after the event; and it did not lead to sustained effort to effect MSC in the priority area identified by patients.

Nevertheless, most participants in the study believed that involvement activities had intrinsic value, facilitating the implementation of MSC. The value attributed to involvement sustained the idea of involvement itself since as the anthropologist Graeber^44^ has remarked, value can be considered as the way in which specific activities are made meaningful to those involved. Investigating how value is produced—and for whom—through involvement might offer a way of rethinking impact assessment in involvement which Edelman and Barron^22^ have faulted for treating this as if it were an intervention in its own right, rather than integral to a larger process. As these authors suggest, rethinking impact requires revisiting the goals and purpose of involvement. This study further suggests a need to identify which goals and purposes are shared by different constituencies as we do not know whether patients and the public who were involved in MSC in London and GM would have recognized the value that emerged from our interviewees’ accounts.
